# Characterization of a biogas-producing microbial community by short-read next generation DNA sequencing

**DOI:** 10.1186/1754-6834-5-41

**Published:** 2012-07-12

**Authors:** Roland Wirth, Etelka Kovács, Gergely Maróti, Zoltán Bagi, Gábor Rákhely, Kornél L Kovács

**Affiliations:** 1 Department of Biotechnology, University of Szeged, Középfasor 52, Szeged, H-6726, Hungary; 2 Institute of Biochemistry, Biological Research Center, Hungarian Academy of Sciences, Temesvári krt. 62, Szeged, H-6726, Hungary; 3 Bay Zoltán Nonprofit Research Ltd, Derkovits fasor 2, Szeged, H-6726, Hungary; 4 Institute of Biophysics, Biological Research Center, Hungarian Academy of Sciences, Temesvári krt. 62, Szeged, H-6726, Hungary

**Keywords:** Biogas, Next-generation sequencing, DNA, Microbial community structure, Bacteria, Methanogens, SOLiD™, Metagenomics, Hydrogen metabolism

## Abstract

**Background:**

Renewable energy production is currently a major issue worldwide. Biogas is a promising renewable energy carrier as the technology of its production combines the elimination of organic waste with the formation of a versatile energy carrier, methane. In consequence of the complexity of the microbial communities and metabolic pathways involved the biotechnology of the microbiological process leading to biogas production is poorly understood. Metagenomic approaches are suitable means of addressing related questions. In the present work a novel high-throughput technique was tested for its benefits in resolving the functional and taxonomical complexity of such microbial consortia.

**Results:**

It was demonstrated that the extremely parallel SOLiD™ short-read DNA sequencing platform is capable of providing sufficient useful information to decipher the systematic and functional contexts within a biogas-producing community. Although this technology has not been employed to address such problems previously, the data obtained compare well with those from similar high-throughput approaches such as 454-pyrosequencing GS FLX or Titanium. The predominant microbes contributing to the decomposition of organic matter include members of the Eubacteria, class Clostridia, order Clostridiales, family Clostridiaceae. Bacteria belonging in other systematic groups contribute to the diversity of the microbial consortium. Archaea comprise a remarkably small minority in this community, given their crucial role in biogas production. Among the Archaea, the predominant order is the Methanomicrobiales and the most abundant species is *Methanoculleus marisnigri*. The Methanomicrobiales are hydrogenotrophic methanogens. Besides corroborating earlier findings on the significance of the contribution of the Clostridia to organic substrate decomposition, the results demonstrate the importance of the metabolism of hydrogen within the biogas producing microbial community.

**Conclusions:**

Both microbiological diversity and the regulatory role of the hydrogen metabolism appear to be the driving forces optimizing biogas-producing microbial communities. The findings may allow a rational design of these communities to promote greater efficacy in large-scale practical systems. The composition of an optimal biogas-producing consortium can be determined through the use of this approach, and this systematic methodology allows the design of the optimal microbial community structure for any biogas plant. In this way, metagenomic studies can contribute to significant progress in the efficacy and economic improvement of biogas production.

## Background

The utilization of fossil fuels on a global scale is limited by the availability of these resources and by the environmental effects of their excessive exploitation. The production of renewable energy carriers is therefore currently receiving increasing attention worldwide. Biogas is a promising candidate as the technology of its production may combine the treatment of various organic wastes with the generation of an energy carrier for the most versatile applications [1-4]. Biogas can be converted to heat and/or electricity, and its purified derivative, biomethane, is suitable for every function for which fossil natural gas is used today. The decomposition of organic materials by a microbial community is carried out under anaerobic conditions [5]. The great variety of diverse microbes that participate in the microbial food chain gradually degrade the complex molecules essentially to a mixture of CH_4_ and CO_2_[6-9]. The actions of the various microbes, involving members of the Eubacteria and Archaea, are coordinated by environmental and internal factors. The composition of this microbial consortium depends on various factors, such as substrate ingredients, temperature, pH, mixing, or the geometry of the anaerobic digester. A clear understanding of the organization and behavior of this multifarious community is crucial for optimization of their performance and attainment of the stable operation of the process. Classical microbiological methods are principally based on studies of isolated pure strains of microbes, and hence are of little help when the goal is elucidation of the relationships among members of a complex microbial consortium in order to improve the overall performance.

The developent of high-throughput sequencing technologies has opened up new avenues for such investigations. Methods with which to reveal the compositions of microbial communities, based on the generation of 16 S rRNA gene clone libraries and Sanger sequencing of the 16 S rDNA amplicons, have recently been devised [10-13]. Archaeal community members have been identified and semi-quantitatively enumerated through the use of the *mcrA* gene, which codes for one of the key enzymes in methanogenesis, the α-subunit of methyl-coenzyme M reductase occurring uniquely in methanogens [14]. Alterations in the organization of methanogenic communities under various conditions have been reported on the basis of this phylogenetic marker [15-19].

The automated Sanger sequencing approach is frequently referred to as “first generation sequencing”. The past few years have brought important technical breakthroughs and the “next-generation sequencing” techniques have been developed. A common feature of these methods, which employ various chemical reactions for the rapid determination of DNA sequences [20,21], is the production of huge databases prepared from relatively short sequence fragments and the use of sophisticated bioinformatics to analyze the results [22]. This metagenomic approach allows the real-time study of live consortia in various environments through identification of the members of these communities [23-25] and/or determination of the relative abundances of particular physiological functions, reflected in the occurrence of specific enzymes [26-28]. Currently the most widespread next-generation sequencing method employs 454-pyrosequencing procedures for metagenomic purposes (Roche). This technique has been used for the characterization of biogas-producing communities [29-33], among numerous other applications. A fundamentally different methodology is offered by the SOLiD™ (sequencing by oligo ligation and detection) technology (Applied Biosystems). As indicated by its name, SOLiD™ is based on a ligation reaction and each nucleotide is interrogated twice, which significantly reduces the potential errors arising from misreading and thereby improves the reliability of the data [34,35]. Since its introduction onto the market in 2007, a number of systems have been investigated with the SOLiD™ method [36-39], but as far as we are aware biogas-producing microbial communities have not been analyzed by SOLiD™ so far. Besides its exceptional accuracy, the fundamental differences as compared with the 454-pyrosequencing approach are the extremely high throughput of the SOLiD system (200 Gb/run) and the short-read technology (50–75 nucleotides/read).

The aim of the present study was to determine the possibility of applying this short-read next-generation sequencing technology to characterize the composite microbial consortium developing in a biogas fermenter and to test whether the results validate those obtained by using the pyrosequencing approach. Samples were taken from an anaerobic fermenter fed primarily with plant biomass and pig manure slurry so that the conclusions could be compared with those drawn from other data sets relating to distinct anaerobic degradation processes with similar substrates.

## Results and discussion

### Distribution of metabolic functions in the microbial community

**Figure 1:**
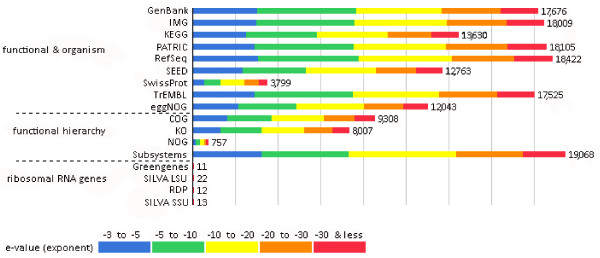
**Source hit distribution.** Legend: The graph displays the number of features in our examined dataset that were annotated by the different databases: GenBank- National Institutes of Health Genetic Sequence Database, IMG- Integrated Microbial Genomes at the Joint Genome Institute, KEGG- Kyoto Encyclopedia of Genes and Genomes, PATRIC- Pathosystems Resource Integration Center, RefSec- National Center for Biotechnology Information Reference Sequences Database, SEED- The SEED Project, SwissProt- Swiss-Prot Uniport Knowledgebase, TrEMBL- TrEMBL Uniport Knowledgebase, eggNOG- evolutionary genealogy of genes: Non-supervised Orthologous Groups, COG- eggNOG: Clusters of Orthologous Groups, KO- KEGG Orthology, NOG- eggNOG: Non-supervised Orthologous Groups, Subsystems- SEDD Subsystem Annotation, Greengenes- 16 S rRNA Gene Database, SILVA LSU- SILVA Large Subunit rRNA Database, RDP- Ribosomal Database Project, SILVA SSU- SILVA Small Subunit rRNA Database. The bars represent annotated reads, which are colored according to their e-value range

**Figure 2:**
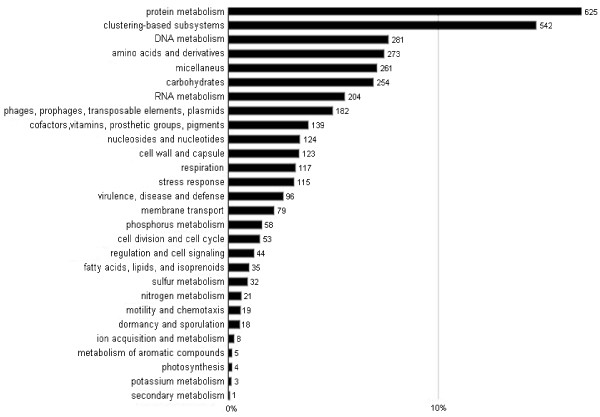
**Functional hierarchical classification analysis.**Legend: The graph shows the abundances of COGs in % using best hits of Subsystems protein database. The most abundant functions are related to biosynthesis, bioenergetics and housekeeping. The numbers on the top of the columns indicate filtered hits, for filtration rules see Material and Methods, data normalization and analysis section

In order to gain an insight into the diverse biochemistry of the biogas-producing community, the short DNA sequences generated by parallel sequencing were used to create environmental gene tags (EGTs) and clusters of orthologous groups of proteins (COGs). The raw sequence reads of about 50 bp were assembled into contigs by using the CLC Bio Genomics Work Bench software [40]. The generated contigs were uploaded to the MG-RAST server, where the data were automatically normalized, processed and evaluated. Those that passed the quality control (see Materials and Methods) were aligned to sequences stored in a number of public databases [41]. This permits classification in the taxonomic and functional hierarchy. Figure 1. reflects the reliability of the results. 26,895 contigs passed the quality control. The contigs were translated into proteins, yielding 13,545 (52%) predicted protein sequences. 12,441 (91%) of the annotated features could be placed in the functional hierarchy. In this way, the DNA sequences from the SOLiD™ reads could be linked to metabolic functions. The results are depicted in Figure 2.

Most of the COGs are linked to information storage and the basic metabolisms of the organic macromolecules (proteins, nucleic acids, lipids, and carbohydrates). Similarly, a large number of COGs related to the biosynthesis of basic cell components, such as cell wall material, vitamins, protective mechanisms and stress responses. These functions are required for the appropriate performance of the community, and therefore are expected to manifest themselves. The high numbers of protein and DNA metabolism COGs suggest that the cells are mostly active. Energy generation and storage are further representations of important functional groups of COGs. These findings are in line with previous studies which indicated that the housekeeping mechanisms and carbohydrate metabolism are predominant. Among the genes involved in the carbohydrate metabolism, those that degrade cellulose are particularly important for the efficient breakdown of the cellulosic biomass substrate. The 16 S rDNA hits and COGs demonstrated that the Firmicutes phylum is of outstanding importance in cellulose degradation by the biogas microbial community, corroborating earlier findings [29-31,42].

### Taxonomic profile of the biogas microbial community

**Figure 3:**
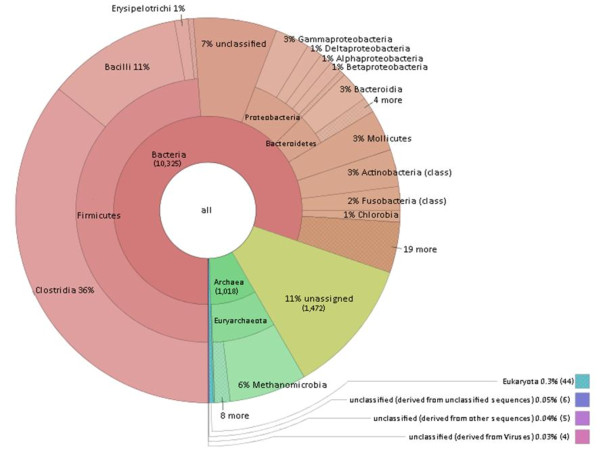
**Taxonomic distribution of the biogas community.** Legend: Allocation of assembled contig sequences to microbial genome. Results were obtained by best M5nr database hits. Bacteria dominate the community, Archaea represent about 10% of the microbiome. Within Firmicutes the Clostridia stand out, among Archaea the hydrogenotrophic methanogens were found in highest number. The numbers in parentheses show the abundances, i.e. the number of sequence features with a hit. The figure was prepared by Krona interactive visualization program (offered by MG-RAST [44])

The assembled contigs were subjected to taxonomic analysis through use of the MG-RAST server [43]. The results were filtered for e-values, percentages of homology and lengths of homology. The ensuing identification and abundance list clearly showed that prokaryotes comprised the most abundant domain; the predominant systematic groups were the Bacteria and Archaea (Figure 3). Within the Bacteria domain, the Firmicutes phylum proved most abundant. The classes Clostridia and Bacilli belonging in this phylum accounted for the majority of the Bacteria in the biogas fermenter. In the Archaea domain, the Methanomicrobiales family provided a preponderance of the identified species. Members of the above-mentioned systematic groups have been identified previously in the anaerobic digestion of maize silage and silage supplemented with animal manure [29-31,42]. It should be noted that a number of sequence reads did not exhibit homology to any of the known and sequenced microbial species, which implies the presence of numerous so far unidentified microbes in biogas fermenters.

### The bacteria domain

More than 1,000 representatives of the Bacteria domain were identified in the metagenomic database.

The first step in the anaerobic degradation of complex organic substrates involves the breakdown of large molecules by hydrolysis [45,46]. Certain communities of bacteria are capable of the efficient hydrolysis of plant biomass rich in lignocellulose. Most of these bacteria belong in the classes of the Clostridia and Bacilli. As expected, the overwhelming majority of the identified abundant species in our biogas fermenter were members of the Clostridia (36%) and Bacilli (11%) classes, together with members of the Bacteroidia (3%), Mollicutes (3%), Gammaproteobacteria (3%) and Actinobacteria (3%) classes (Figure 3). Unassigned and unidentified sequences were ignored in this analysis. The most abundant identified species are listed in Table 1. and the presence or absence of cellulose degrading activity and hydrogenase enzymes is indicated.

**Table 1:** The 40 most frequently found microbial species in the Bacteria domain

**Phylum**	**Class**	**Species**	**Abundance**	**Cellulase**	**H2 production**
Firmicutes	Clostridia	*Clostridium thermocellum*	406	+	+
Firmicutes	Clostridia	*Alkaliphilus metalliredigens*	310	-	+
Firmicutes	Clostridia	*Desulfitobacterium hafniense*	246	-	+
Firmicutes	Clostridia	*Caldanaerobacter subterraneus*	237	-	+
Firmicutes	Clostridia	*Pelotomaculum thermopropionicum*	227	-	+
Firmicutes	Clostridia	*Finegoldia magna*	208	-	+
Firmicutes	Clostridia	*Syntrophomonas wolfei*	203	-	+
Firmicutes	Clostridia	*Clostridium difficile*	188	+	+
Firmicutes	Clostridia	*Moorella thermoacetica*	186	-	+
Firmicutes	Clostridia	*Clostridium kluyveri*	176	n.d.	+
Firmicutes	Clostridia	*Carboxydothermus hydrogenoformans*	161	-	+
Firmicutes	Clostridia	*Heliobacterium modesticaldum*	150	-	+
Firmicutes	Clostridia	*Desulfotomaculum reducens*	139	-	+
Firmicutes	Clostridia	*Clostridium cellulolyticum*	126	+	+
Firmicutes	Bacilli	*Enterococcus faecalis*	112	n.d.	+
Firmicutes	Bacilli	*Bacillus cereus*	102	+	+
Firmicutes	Bacilli	*Streptococcus suis*	93	+	-
Firmicutes	Clostridia	*Caldicellulosiruptor saccharolyticus*	93	+	+
Firmicutes	Clostridia	*Clostridium perfringens*	87	-	+
Firmicutes	Clostridia	*Thermoanaerobacterium thermosaccharolyticum*	86	-	+
Firmicutes	Clostridia	*Ruminococcus albus*	83	+	+
Firmicutes	Clostridia	*Clostridium saccharolyticum*	78	+	+
Firmicutes	Clostridia	*Clostridium acetobutylicum*	63	+	+
Firmicutes	Bacilli	*Bacillus thuringiensis*	62	+	+
Firmicutes	Bacilli	*Streptococcus pneumoniae*	61	-	n.d.
Firmicutes	Bacilli	*Listeria monocytogenes*	61	n.d.	n.d.
Firmicutes	Bacilli	*Streptococcus agalactiae*	57	n.d.	-
Firmicutes	Bacilli	*Enterococcus faecium*	57	n.d.	+
Firmicutes	Clostridia	*Anaerotruncus colihominis*	55	n.d.	n.d.
Firmicutes	Clostridia	*Faecalibacterium prausnitzii*	54	-	+
Firmicutes	Clostridia	*Clostridium carboxidivorans*	49	-	+
Firmicutes	Bacilli	*Staphylococcus epidermidis*	44	+	+
Bacteroidetes	Bacteroidia	*Bacteroides capillosus*	73	+	+
Bacteroidetes	Bacteroidia	*Bacteroides thetaiotaomicron*	46	+	+
Bacteroidetes	Bacteroidia	*Parabacteroides distasonis*	46	-	+
Tenericutes	Mollicutes	*Acholeplasma laidlawii*	247	-	-
Proteabacteria	Gammaproteobacteria	*Escherichia coli*	23	-	+
Actinobacteria	Actinobacteria (class)	*Slackia heliotrinireducens*	70	-	+
Actinobacteria	Actinobacteria (class)	*Bifidobacterium longum*	46	-	-
Unclassified (derived from Bacteria)	Unclassified (derived from Bacteria)	*Candidatus Cloacamonas acidaminovorans*	889	-	+

Results were based on best M5nr database hits. The relative abundance values and presence of cellulose or hydrogenase activities are indicated. n.d. = not determined, i.e., no information was found in the protein databases or indicated as hypothetical protein.

Among the Clostridia, *Clostridium thermocellum* occurred most frequently. This species can hydrolyze cellulose efficiently by means of its extracellular cellulases, which are organized into cellulosomes [47,48]. An outstanding member of this class is *C. kluyveri*, which is unique among the Clostridia, because it uses ethanol and acetate as sole energy sources and converts these substrates to butyrate and H_2_[49]. A prominent and well-characterized species is *C. acetobutylicum*, which exerts cellulolytic, saccharolytic and H_2_-producing activities. The fermentation pathways may yield organic acids such as acetate and butyrate (acetogenesis), or acetone, butanol and ethanol (solventogenesis) [50,51]. *C. perfingens* generates lactate, acetate and butyrate from sugars, and through its [FeFe]-hydrogenase, it can also produce H_2_[52]. Similarly to *C. thermocellumC. cellulolyticum* is a well-known strain that degrades cellulose to acetate and evolves CO_2_ and H_2_[53]. *C. saccharolyticum* additionally possesses cellulolytic activity. The fermentation products include acetate, ethanol, H_2_ and CO_2_[54]. *C. difficile* is one of the rare pathogens [55] found in a biogas community. *Thermoanaerobacterium thermosaccharolyticum* is a H_2_-producing bacterium that has been reported to live in co-culture with *C. thermocellum*, the mixed culture producing more H_2_ than the pure cultures [56,57]. *Ruminococcus albus* has been noted for its efficient cellulose-degrading activity by cellulosomes; the major fermentation product is ethanol [58]. Both *Anaerotruncus colihominis* and *Faecalibacterium prausnitzki* colonize the intestine and produce various volatile organic acids from glucose and acetate, respectively [59,60].

Besides being capable of reductive dechlorination, *Desulfitobacterium hafniense* can produce sulfide from thiosulfate or sulfite, but cannot reduce sulfate. As carbon source it prefers to ferment pyruvate and lactate. This species is also known to contain Hup (hydrogen-uptake) type of [NiFe]-hydrogenases [61]. *Heliobacterium modesticalum* can grow in either photoheterotrophic or chemotrophic mode. Under chemotrophic conditions it ferments acetate to H_2_ and CO_2_. It also contains a number of hydrogenases, including [NiFe]- and [FeFe]-hydrogenases [62]. H_2_ and acetate are generated by *Caldanaerobacter subterraneus* from lactose, glucose or cellobiose as substrate [63]. *Syntrophomonas wolferi* ferments long-chain fatty acids and lives in co-culture with methanogenic Archaea [64]. *Pelotomaculum thermopropionicum* too forms a syntrophic relationship with methanogens, and its abundance in the anaerobic digester community is therefore reasonable. The syntrophic associations play important roles in efficient biogas formation [65]. The unique members of the Clostridia class, *Alkaliphilus metalliredigens* and *Desulfotomaculum reducens*, were detected in unexpectedly high amounts. These bacteria are known to use lactate and acetate as electron sources for the reduction of iron and cobalt in anaerobic respiration [66]. Although it may not be trivial to explain the occurrence of metal-reducing bacteria in an anaerobic biogas-producing community, it should be noted that these bacteria also possess highly active [FeFe]-hydrogenases [67]. *Caldicellulosiruptor saccharolyticus* is a cellulose-degrading and H_2_-producing bacterium. Addition of a pure culture of *C. saccharolyticus* to sewage sludge, plant biomass, animal manure or a mixture of these significantly increased the extent of biogas production [68]. *Finegoldia magna* has noteworthy substrate specificity, as it can utilize only fructose from among a range of sugars, and produces acetate [69]. *F. magna* also carries the genes for a putative hydrogenase [70]. The large number and proportion of members of the Clostridiales order are indicative of the important role of these bacteria in the proper functioning of the microbial community in an anaerobic digester fed with complex substrates. Their contribution to the breakdown of polysaccharide molecules may be explained by the high cellulolytic activity of numerous members of the Clostridiales order, and members of the Clostridiaceae family are capable of performing diverse fermentation pathways. They primarily ferment sugars to organic acids [71]. The Wood-Ljungdahl pathway, also known as the reductive acetyl-CoA pathway, plays an important role in this process, which is typical in acetogenic bacteria and in some Archaea [72]. In this process, CO_2_ is reduced to CO and then converted to acetyl-CoA, H_2_ serving as electron donor [73]. In the anaerobic digester, the aceticlastic Archaea split acetate to CH_4_ and CO_2_ in an energy gaining process [74]. Besides the acetogenic Clostridia discussed above, *Moorella thermoacetica* and *Carboxidothermus hydrogenoformans* also obtain energy via the Wood-Ljungdahl pathway. It should additionally be noted that a large number of Clostridia actively produce H_2_, an important substrate for the hydrogenotrophic methanogens. It is noteworthy that *Cr. hydrogenoformans* is able to use CO as carbon source as electron donor and water as an electron acceptor, to produce acetate and H_2_[75,76]. Both *Cr. hydrogenoformans* and *M. thermoacetica* are capable of H_2_ production [77]. The predominance of the Clostridia in the anaerobic digester community triggers the activity of the hydrogenotrophic methanogens, which must keep the H_2_ partial pressure in the system low in order to ensure system stability [78]. The delicate balance between the Clostridia and hydrogenotrophic methanogens must be a determining factor within the biogas-producing microbial consortium (Figure 3).

The second largest group of bacteria in the anaerobic degradation community is the class of Bacilli in the Bacteria domain. The most abundant species from this class in our fermenters was *Enterococcus faecalis*. This strain, an anaerobic Gram-positive bacterium found in the digestive system, is able to hydrolyze plant polysaccharides and possesses hydrogenase activity in its formate dehydrogenase complex [79]. *E. faecium* is also common in the gastrointestinal system. These microbes convert carbohydrates such as fructose, maltose, lactose and galactose to acetate and ethanol [80,81]. *Bacillus cereus* and *B. thuringiensis* can carry out both aerobic and anaerobic metabolism. Under anaerobic conditions, *B. cereus* ferments glucose to a mixture of acetate, lactate and ethanol, while *B. thuringiensis* produces mostly lactate [82,83]. *Streptococcus pneumonia* is a pathogen that converts glucose to lactate [84]. Its relative *S. suis* can ferment glucose, lactose, maltose and trehalose to a mixture of volatile fatty acids [85], while *S. agalactiae* also generates ethanol beside the volatile acids [86,87]. Additional pathogenic Bacilli detected in the anaerobic digester community, though in low abundance, include *Staphylococcus epidermis* and *Listeria monocytogenes*[88].

Over and above the members of the Clostridia and Bacilli classes discussed above, the study revealed additional members of the microbial systematic groups in the biogas-producing community, though their contribution to the microbiological food chain is probably limited relative to that of the Clostridia and Bacilli. Bacteroidia species were identified in meaningful quantities. Members of the Bacteriodia are common in nature at sites where degradable organic material is to be found, such as plants and other forms of biomass. *Bacteroides capillosus* is an intestinal bacterium that ferments lactate and produces H_2_, and also displays cellulolytic activity [89]. As an outstanding example of human-bacterium symbiosis, *Bacteroides thetaiotamicron* is a constituent of the intestinal flora, which specializes in hydrolyzing polysaccharides of plant origin, *i.e.* cellulose and starch, as carbon sources [90,91]. *Parabacteroides distasionis* is a Gram-negative, non-spore-forming bacterium that produces volatile organic acids [92].

The members of the Mollicutes are facultative anaerobes. Under anaerobic conditions, they produce organic acids, which may be utilized by the acidoclastic methanogens [93]. Acoleplasmatales is the most abundant among the relatively few Mollicutes class members. *Acoleplasma laidlawii* ferments glucose to produce lactic acid, saturated fatty acids and acetate [94]. All these fermentation products are subsequently converted to biogas by the acetoclastic Archaea in the methanogenic consortium. Although Gammaproteobacteria are frequently found in diverse habitats, they do not appear to dominate in the biogas-producing community. *Escherichia coli*, one of the most widespread and certainly the most thoroughly studied bacterium, was present in the anaerobic community. *E. coli*, a facultative anaerobe, has a highly versatile metabolism. Under anaerobic conditions, it produces lactate, succinate, ethanol, acetate, H_2_ and CO_2_ in a mixed acid fermentation [95]. Various [NiFe]-hydrogenases are involved in the metabolism of H_2 ,_ and a syntrophic relationship often develops with H_2_ consumers in order to keep the H_2_ partial pressure low in the entire system [96]. Members of the Actinobacteria class are commonly found in soils and natural waters. Some of them effectively break down complex organic material such as cellulose, and thereby play an important role in the carbon cycle [97]. Furthermore, members of this group are known to produce lignin-degrading enzymes [98]. Two species of Actinobacteria were identified in our biogas fermenter samples: *Slackia heliotrinireducens* and *Bifidobacterium longum. Sl. heliotrinireducens* is a Gram-positive anaerobic bacterium which can reduce nitrate to ammonia if there are electron donors (H_2_ or formate) in the system. This organism has also been reported to produce acetic acid and lactic acid, and contains a hydrogenase [99,100]. *Bf. longum* is a Gram-positive bacterium found as a symbiont in the human normal intestinal flora [101]. It metabolizes oligosaccharides and releases lactic acid, which helps control the normal microflora.

In addition to the known phylogenetic categories, 7% of the sequences belong to the Bacteria domain, but lacks detailed classification. In this group *candidatus Cloacamonas acidaminovorans* was found in remarkably high abundance. This species was also identified in several anaerobic digester microflora [31,102]. *c. Cm. acidaminovorans* gains energy from sugars in the Embden-Meyerhof pathway and from the fermentation of amino acids. It is a fermentative H_2_ producer, containing a [FeFe]-hydrogenase, which is an indication of syntrophic metabolism [103].

### The archaea domain

The volatile organic acids, CO_2_ and H_2_ generated by the acetogens are the substrates of methanogenesis carried out by special Archaea [104,105]. Aceticlastic and hydrogenotrophic methanogens are distinguished in biogas fermentors [106]. The hydrogenotrophic Archaea are capable of reducing CO_2_ to CH_4_, H_2_ being used as an electron donor. The CO_2_-reducing pathway starts with the formation of *N*-carboxymethanofuran from CO_2_ and the C1-carrier methanofuran, which is subsequently reduced to formyl-methanofuran. The reductant is provided from reduced F_420_ (8-hydroxy-5-deazaflavin) and hydrogenases. The central electron carrier in hydrogenotrophic methanogenesis is coenzyme F_420_[107]. As the first step in the inverse Wood-Ljungdahl pathway, acetate is activated to acetyl-CoA with the participation of phosphotransacetylase and acetate kinase in acetotrophs [108]. Carbon monoxide dehydrogenase (CODH) then breaks down acetyl-CoA to CO, a methyl group and CoA [109]. CO is oxidized to CO_2_, which generates the electrons for reduction of the methyl radical to CH_4_[110].

**Figure 4:**
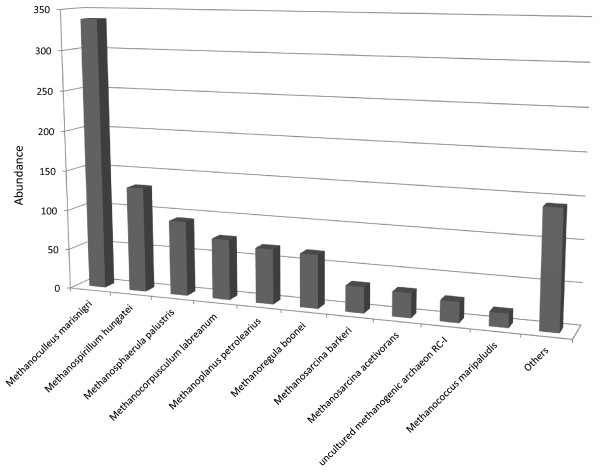
**Most abundant Archaea strains.** Legend: Identification was based on M5nr database. At species level the hydrogenotrophic methanogens dominate. Acetotrophic methanogens show relatively low representation in the biogas community

**Figure 5:**
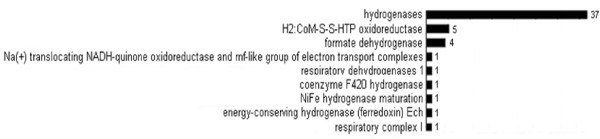
**Energy and hydrogen metabolism related enzyme functions in the biogas producing community.** Legend: The results were extracted from the Subsystem database. The numbers on the top of the columns indicate filtered hits, for filtration rules see Material and Methods, data normalization and analysis section

Around 10% of the identified microbes in the biogas-producing community belonged in the Archaea (Figures 3 and 4). This correlated well with findings in previous studies [30,42]. In the domain of the Archaea the Methanomicrobiales order predominates in the community. Within this order, the most abundant species *is Methanoculleus marisnigri*[111]. Interestingly, the same Archeon has been found in several methanogenic consortia [112,113]. *M. marisnigri* JR1 is the only member of the Methanoculleus genus, which has been sequenced so far [114], and it cannot be excluded that several members of the same genus produce the high abundance of *Methanoculleus*-related reads [42]. Besides *Methanoculleus*, other representatives of Methanomicrobiales contribute to the plethora of hydrogenotrophic methanogens, *e.g. Methanospirillum hungatei*[115], *Methanosphaerula palustris*[116], *Methanoregula boonei*[117], *Methanocorpusculum labreanum*[118] and *Methanoplanus petrolearius*[119]. From the class of Methanococci*, Methanococcus maripalidus* is also a hydrogenotrophic methanogen [120] (Figure 5). Among the aceticlastic methanogens, *Methanosarcina acetivorans*[121] was present in a relative majority. An unidentified archaeon detected among rice rhizophere methanogens was also found in the anaerobic biogas community. This species was described as having a unique aerotolerant H_2_/CO_2_ dependent lifestyle and enzymes for carbohydrate metabolism and assimilatory sulfate reduction [122].

The predominance of the hydrogenotrophic methanogens strongly suggests that methane is generated mainly by the hydrogenotrophic pathway and aceticlastic methanogenesis plays a secondary role in the anaerobic digestion process (Figures 3 and 4.). H_2_ is produced for the hydrogenotrophic methanogens by the acetogens, *e.g.* Clostridia as shown above, or by syntrophic acetate oxidation [103,123,124]. At any rate the close proximity of the participating microbes and the very delicately balanced H_2_ metabolism are a must in these communities in order to keep the H_2_ concentration low and favor CH_4_ formation [68,106]. Acetate stimulates the growth of *Methanospirillum hungatei*[115], *Methanosphaerula. palustris*[117], *Methanoregula boonei*[118], *Methanocorpusculum labreanum*[118]*, Methanococcus maripalidus*[118] and *Methanoplanus petrolearius*[119]. In contrast, *Methanoculleus marisnigri* can only use CO_2_ as carbon source [110]. Accordingly, adequate acetate supply is required for the growth of hydrogenotrophic and aceticlastic methanogenesis and syntrophic acetate oxidizers [103,118,119,121].

All of the identified Methanomicrobiales possess H_2_-activating membrane-associated hydrogenases [42,117,119,125], and the relative wealth of hydrogenase-specific DNA reads corroborates the importance of these enzymes in the anaerobic degradation of organic material (Table 1 and Figure 5). Although the contributions of Eubacteria and Archaea cannot be distinguished in Figure 5, the widespread presence of H_2_-activating enzymes underlines their importance in the physiology of the biogas-producing community. A highly efficient interspecies H_2_ transfer [126] must take place between the H_2_-forming and consuming partners.

Besides the hydrogenases other genes encoding important redox proteins and likely to be connected to H_2_ metabolism were detected in the biogas fermenter, *e.g.* coenzyme M heterodisulfide heptanyl threonine phosphate (CoM-S-S-HTP) oxidoreductase, formate dehydrogenase and coenzyme F_420_ hydrogenase. CoM-S-S-HTP oxidoreductase catalyzes the conversion of CoM-S-S-HTP to HS-HTP (7-mercaptoheptanyl-*L*-threonine phosphate), which is a unique methanogenic cofactor in all methanogens [127]. Formate dehydrogenase extracts the hydrogen from formate and releases CO_2_[128]. Reduced F_420_ is oxidized by a membrane bound electron transport system. When F_420_ is oxidized, an equimolar amount of CoM-S-S-HTP is reduced. CoM-S-S-HTP oxidoreductase is common in all methanogens but formate dehydrogenase and coenzyme F_420_ are only typical to hydrogenotrophic methanogens [108].

### Comparison of the 454-pyrosequencing and SOLiD™ metagenomic results

Previous studies designed to improve the understanding of microbial communities in biogas-producing anaerobic digestors, based on next-generation sequencing methods, relied exclusively on the pyrosequencing technique [29-31,42]. The substrates fed into the fermentors included animal manure and green plant biomass (maize or green rye silage), commonly employed in German biogas facilities. Our laboratory fermenters were fed with a substrate mix with a similar composition, but our operational parameters, sample handling, DNA extraction protocols and sequence data collection and analysis methods were different.

The SOLiD™ sequencing method produces short individual reads (50 nucleotides) in a significantly higher number than does pyrosequencing. We have generated and analyzed 23,897,590 individual reads representing 1,194,879,500 bases. In previous studies, two versions of 454-pyrosequencing were employed and compared: GS FLX and Titanium [12]. The latter provides somewhat longer reads and increased throughput relative to GS FLX (454 GS FLX resulted in 616,072 sequence reads with an average read length of 230 bases, while Titanium resulted in 1,347,644 reads with an average read length of 368 bases). As a general rule of thumb, the longer the read sequence and the higher the number of independent reads, the more reliable the data.

**Figure 6:**
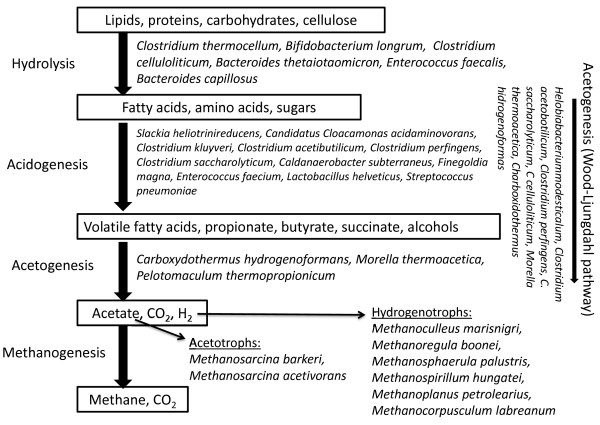
**The most abundant members of the biogas producing food-chain.** Legend: The identified microbes are arranged according to their known physiological roles in the steps of the anaerobic degradation process. For detailed explanation see text

In a comparison of the Bacteria domain, a remarkably good match was found between the data sets obtained by the various next-generation sequencing methods. In all cases, the class Clostridia comprised the most widespread group of microbes in the biogas fermenters. The Clostridia are noted for their highly effective cellulose degradation potential [129], and are therefore essential in the breakdown of lignocellulosic substrates in the biogas process. It should also be noted that the majority of Clostridia possess highly active hydrogenases. This is in line with the observation that hydrogenases have been found in large quantity among the redox enzymes in the biogas producing community (Figure 5.). Thus, the Clostridia may contribute to the widening of at least two bottlenecks in the biogas process, through the hydrolysis of large polymeric substrates and the *in situ* production of H_2_, an important reductant for the hydrogenotrophic methanogens [70,130]. The positions of the most abundant strains in the methanogenic microbial food chain are summarized in Figure 6.

At the level of resolution of the abundances of individual strains, the most frequently occurring species likewise displayed a good correlation. Strains noted for their highly efficient polysaccharide degradation capabilities, such as *Clostridium thermocellum*, *C. cellulolyticum* and *Caldicellulosiruptor saccharolyticus*, are found to be the most abundant, regardless of the sequencing method used for their identification.

Similarly to the Bacteria, the members of the Archaea domain demonstrate a markedly comparable community structure, which is clearly reflected in any next-generation sequencing dataset. The analysis of the data at the species level revealed a strong correlation between the findings of the 454-pyrosequencing and SOLiD™ next-generation sequencing technology platforms. The Methanomicrobiales were indicated to constitute the majority of the Archaea in this environment by the sequencing with the 454 GS FLX [29-31], 454 Titanium [42] and SOLiD™ platforms alike. Within this taxon, the predominant genus is Methanoculleus, and the most abundant species according to our SOLiD™ results is *M. marisnigri*. Exactly the same picture was revealed by the 454-pyrosequencing approach [29-31,42]. It is worth noting that the Methanomicrobiales are hydrogenotrophic methanogens, which are capable of reducing CO_2_ with H_2_ to produce additional CH_4_ in the biogas-producing consortium. The DNA-based community structure analysis of anaerobic degradation samples has already demonstrated the enormous importance of hydrogenotrophic methanogens.

## Conclusions

The metagenomic analysis of biogas-producing microbial communities is a novel approach by which to study the complex interaction among microbes in an environment that is important for both basic research and the practical aspects of improvement of renewable energy production from biomass. In the present study, the Applied Biosystems’ SOLiD™ sequencing platform was used to collect relevant data. This next-generation DNA sequencing approach has not been used previously to characterize the microbial consortium of a biogas fermentor. Similar data sets determined with the Roche 454-pyrosequencer have been analyzed and reported [29-31,42]. SOLiD™ differs from the 454 technique in several important technical aspects. SOLiD™ sequencing is based on ligation reactions, operates with a short read length and a much higher throughput than that of the 454 technique, and each nucleotide is read twice by the system, which makes the data highly accurate. Metagenomics is a special application and poses a real challenge since the complexity of the samples requires both high throughput and long reads. It is therefore important to compare the results obtained on a similar microbial community by using different analytical approaches; this can validate the various methodologies. It should be emphasized that a contribution is also made by microbes that are unknown or undetermined in the databases. These are not available for study by any of the current methods, but the rapid increase in available genome information justifies the exploitation of novel, high-throughput genomic methods in the field of community analysis.

One conclusion drawn from this study is that the sets of metagenomic information deduced from the databases via the various methods correlate well with each other. In this way, the databases generated through use of either of the investigated next-generation sequencing approaches have been validated and appear reliable and reproducible.

Although the anaerobic fermentation conditions (fermenter size, feedstock composition and origin, mixing, inoculum composition, *etc*.) were somewhat different, the SOLiD™ and 454-pyrosequencing data appear to lead to the same fundamental conclusions. Members of the Firmicutes and Bacteroides phyla play the most important role in the hydrolysis of the plant biomass and in the secondary fermentation. In particular, many *Clostridium* species were identified which possess cellulolytic and H_2_-producing activities, both properties probably being essential for the efficient degradation of the biomass. In the Archaea domain, Methanomicrobiales is the most abundant order that uses CO_2_ as a carbon source and H_2_ as an electron donor for methanogenesis. The predominance of the Methanomicrobiales and many hydrogenases suggests that the hydrogenotrophic pathway leading to CH_4_ formation may be more significant than recognized earlier [131-134]. *Methanoculleus marisnigri* proved to be the principal species among the archaeal habitants in the biogas fermenter. Interestingly, the same Archaeon has been identified as the most abundant in an anaerobic digester operated under different conditions [29-31,42,113,114]. It is therefore concluded that an optimized balance between H_2_ producers and consumers is critical for the efficient operation of the biogas microbial community.

## Methods

### Fermentation conditions

The anaerobic digestion experiments were performed in 6-liter, continuously stirred tank reactors with a working volume of 5 liters. The fermenters were designed and constructed by Biospin Ltd, Hungary and installed at the Department of Biotechnology, University of Szeged [135]. The reactors were fed periodically with maize silage (68% oTS) added to pig manure slurry to sustain an average 15% oTS. Mixing of three fermenters operated in parallel was achieved with a single electronic engine through belt transmission in order to maintain identical mixing conditions. Heating was maintained by an electronically heated jacket which surrounded the cylindrical apparatus. Temperature was measured with a bimetallic-type sensor, and was maintained constant at 37 ± 1.0 °C. Electrodes for continuous monitoring of pH and redox potential were inserted into the fermentor in sealed sockets. The evolved gas left the fermentor through flexible neoprene tubing connected to the top plate, where ports for gas sampling through silicone rubber septa were also installed. Gas volume was measured with thermal mass flow controllers (DMFC, Brooks) attached to each gas exit port. The hydraulic retention time 60 days. The pH was maintained between 7.9-8.4. Acetate concentration was 0.1 g/mL, The volatile fatty acid content varied between 1.5 and 1.6 g HAceq/L, the buffering capacity was 9.21-9.28 g CaCO_3_/L. Data were collected, stored and analyzed with special software developed by Merat Ltd., Hungary. The key parameters (temperature, mixing speed and pH) were controlled continuously by the software. Biogas production was 610 L_N_/ kg oTS (organic total solids) with 52% methane content.

### Purification of total DNA from biogas fermenter

A 2-ml liquid fermentation sample was utilized to prepare total community DNA by applying a CTAB based DNA extraction buffer [136-138]. Cell lysis was carried out at 55 °C overnight. Phenol:chloroform (1:1) was used to extract contamination, and the genomic DNA was precipitated with ethanol (90%). The DNA pellet was resuspended in 100 μl of TE buffer [139]. Its quantity was determined in a NanoDrop ND-1000 spectrophotometer (NanoDrop Technologies, Washington, USA). DNA purity was tested by agarose gelelectrophoresis. This method yielded a pure (A_260_/A_280_ = 1.8) and sufficient amount of total DNA (200–800 ng/μl).

### Sequencing the DNA of the biogas fermenting microbial community

Sequencing was performing using an Applied Biosystems SOLiD™ 4 sequencing platform. Primary data analysis was carried out with software provided by the supplier (base-calling). The 50 nucleotide reads were analyzed, quality values for each nucleotide were determined, and the reads were assembled into contigs through use of the CLC Bio Genomics Workbench 4.6 program [40]. The preset parameters were as follows: minimum contig length = 200, similarity = 0.8, length fraction = 0.5, insertion cost = 3, deletion cost = 3, mismatch cost = 2, color space elignment = yes, color error cost = 3.

**Figure 7:**
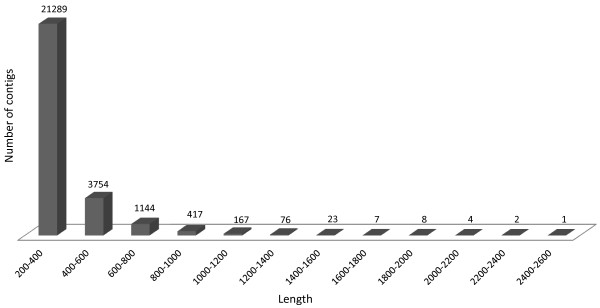
**Contig length distribution.** Legend: The number of contigs generated by CLC bio de novo assembly softvare and falling into the various lenght ranges are plotted. The parameter settings are given in the text

In the contig assembly process, 288 large contigs containing more than 1,000 bp were identified. The average length of the assembled contigs was 333 bp. The cumulative number of all contigs was 26,892, which amassed 8,978,367 bp. The contig size distribution is presented in Figure 7.

### Data normalization and analysis

The assembled contigs were further analyzed by using the MG-RAST software package [140], which is a modified version of RAST (Rapid Annotations based on Subsystem Technology).

The MG-RAST server initially runs a quality control test. If the data appear reliable, the system automatically screens for sequences of potential protein encoding regions (PEGs) via a BLASTX [141] search against the SEED comprehensive non-redundant database compiled from various publicly available sequencing centers and other sources [142]. These databases include several rDNA datasets too, *e.g.* GREENGENES [143], RDP II [144], and European 16 S RNA [145], among other information sources. To identify the gene content of the biogas reactor, all contigs were functionally annotated by means of the clusters of orthologous groups (COGs) of proteins made automatically by the MG-RAST server using the eggnog and COG databases. The generated matches to external databases were used to compute the derived data. The phylogenetic reconstruction of the contig sets was performed by using both the phylogenetic information contained in the SEED nr database and the similarities to the ribosomal RNA database. Functional classifications of the PEGs were computed by projecting against SEED FIGfams [146] and subsystems based on these similarity searches [142]. These functional assignments served as the raw input for an automatically generated initial metabolic reconstruction. The user interface provided a means of altering some of the parameters employed for the functional and metabolic reconstruction computation [140]. The acceptable percentage of identity was set to be >70%, the minimum read length was >35 nucleotides and the e-value cut-off was <10^-6^. The contigs formed from the sequence reads were compared with the M5nr database for phylogenetic analyses [147], which integrated the previously mentioned databases into a single, searchable database offered by MG-RAST.

## Abbreviations

SOLiD™, Sequencing by Oligo Ligation and Detection new generation sequencing platform; CTAB, Cetyltrimethylammonium bromide; EGT, Environmental gene tags; COG, Orthologous group of proteins; CoM-S-S-HTP, Coenzyme M heterodisulfide heptanyl threonine phosphate) oxidoreductase; HS-HTP, 7-mercaptoheptanyl-L-threonine phosphate; PEGs, Potential protein encoding genes; TE buffer, Tris-EDTA buffer (10 mM Tris, 1 mM EDTA pH: 8.0).

## Competing interest

The authors declare that they have no competing interests.

## Authors’ contributions

RW developed the DNA extraction protocol and participated in the evaluation of the data. EK carried out the anaerobic digestion experiments and supervised the operation of the fermenters. GM organized and performed the next-generation sequencing and took part in the data analysis. BZ participated in the experimental work and its design. GR contributed to the data interpretation. KLK conceived the study, participated in its design and compiled the manuscript. All the authors have read and approved the final manuscript.

## Authors’ information

GM is Head of the Metagenomics Laboratory, Bay Zoltán Nonprofit Research Ltd., Szeged, Hungary. RW and EK are PhD students, ZB is a postdoc, RG is an Associate Professor, and KLK is a Full Professor and Department Chairman at the Department of Biotechnology, University of Szeged, Hungary. RG is the Director of the Environmental Research Institute at the University of Szeged. KLK is a Senior Adviser at the Institute of Biophysics, Biological Research Center, Hungarian academy of Sciences and also serves as President of the Hungarian Biogas Association.
